# Single-Use Disposable Waste Upcycling via Thermochemical Conversion Pathway

**DOI:** 10.3390/polym13162617

**Published:** 2021-08-06

**Authors:** Junghee Joo, Seonho Lee, Heeyoung Choi, Kun-Yi Andrew Lin, Jechan Lee

**Affiliations:** 1 Department of Energy Systems Research, Ajou University, 206 World cup-ro, Suwon 16499, Korea; jurno@ajou.ac.kr; 2 Department of Environmental and Safety Engineering, Ajou University, 206 World cup-ro, Suwon 16499, Korea; idosunho99@ajou.ac.kr (S.L.); chk6788@ajou.ac.kr (H.C.); 3 Innovation and Development Center of Sustainable Agriculture, Department of Environmental Engineering, National Chung Hsing University, 250 Kuo-Kuang Road, Taichung 402, Taiwan; linky@nchu.edu.tw

**Keywords:** municipal solid waste, plastic waste, recycling, thermochemical process, waste-to-energy

## Abstract

Herein, the pyrolysis of two types of single-use disposable waste (single-use food containers and corrugated fiberboard) was investigated as an approach to cleanly dispose of municipal solid waste, including plastic waste. For the pyrolysis of single-use food containers or corrugated fiberboard, an increase in temperature tended to increase the yield of pyrolytic gas (i.e., non-condensable gases) and decrease the yield of pyrolytic liquid (i.e., a mixture of condensable compounds) and solid residue. The single-use food container-derived pyrolytic product was largely composed of hydrocarbons with a wide range of carbon numbers from C_1_ to C_32_, while the corrugated fiberboard-derived pyrolytic product was composed of a variety of chemical groups such as phenolic compounds, polycyclic aromatic compounds, and oxygenates involving alcohols, acids, aldehydes, ketones, acetates, and esters. Changes in the pyrolysis temperature from 500 °C to 900 °C had no significant effect on the selectivity toward each chemical group found in the pyrolytic liquid derived from either the single-use food containers or corrugated fiberboard. The co-pyrolysis of the single-use food containers and corrugated fiberboard led to 6 times higher hydrogen (H_2_) selectivity than the pyrolysis of the single-use food containers only. Furthermore, the co-pyrolysis did not form phenolic compounds or polycyclic aromatic compounds that are hazardous environmental pollutants (0% selectivity), indicating that the co-pyrolysis process is an eco-friendly method to treat single-use disposable waste.

## 1. Introduction

The COVID-19 pandemic has led to the widespread use of single-use disposable items such as plastics (e.g., food containers) and paper products for packages and parcels (e.g., corrugated fiberboard). According to United States Environmental Protection Agency, approximately 82 million tons of disposable containers and packaging products were generated in 2018 in the US [1]. The management of single-use products that are not readily biodegradable and unrecyclable is critically important because their mismanagement causes severe economic, environmental, and health concerns [2]. Among used single-use disposable items, only about 50% were recycled, and the others were landfilled or combusted [1]. The recycling of the waste is intended in many countries in various industries such as the sugar industry [3], the building material industry [4], and the material treatment industry [5]. However, most recyclable items end up being rejected at local or regional waste facilities because of high levels of unrecyclable items or other matter such as food waste ending up in the recycling streams (i.e., difficulty in complete sorting or isolating recyclable materials) [6]. Non-recyclable and not-recycled waste can cause big environmental issues such as microplastics [7], nanoplastics [8], landfills filling up fast, the release of greenhouse gases, and toxins leaching into soil and groundwater [9]. This necessitates the development of alternative solutions for recovering value-added products from single-use containers and packaging products such as disposable food containers and corrugated fiberboard in order to eliminate environmental risks.

Pyrolysis is a thermochemical process conducted under an oxygen-limited conditions [10] that is extensively used to recover energy or other resources from various waste materials such as sewage sludge [11], livestock manure [12], food waste [13], everyday waste [14], and plastic waste [15]. The pyrolysis process often leads to liquid oil products containing compounds with large carbon chains [16]. Pyrolysis oil is of low quality because it has a low octane number and contains solid residues [17] and inorganic impurities such as nitrogen, sulfur, phosphorus, and chlorine [18]. To tackle this issue, the co-pyrolysis of two different feedstocks (one with high H/C and low O/C ratios, i.e., plastic waste) has become a topic of interest [19]. For example, the co-pyrolysis of plastic waste and biomass brings about synergistic effects that not only lead to improving the quality and uniformity of pyrolytic products [20] but also to minimizing coke formation [21]. In addition, co-pyrolysis offers an attractive way to minimize the need for waste separation for mixed waste [22].

Even though the literature is rich on the co-pyrolysis of various types of waste materials, there is a gap for single-use food containers and corrugated fiberboard that we aim to address with this study. Based on the reported findings in earlier literature (Table 1), it was hypothesized that the co-pyrolysis process is a viable option for upcycling different types of single-use disposable waste. Through this study, we aimed to investigate the effect of the co-pyrolysis of disposable food containers/corrugated fiberboard on pyrolytic product distribution in terms of yield and selectivity by comparing the co-pyrolysis with the pyrolysis of single feedstock. The influence of co-pyrolysis parameters such as temperature and feedstock composition on pyrolytic product properties was explored. The results of this investigation should aid in evaluating the application of co-pyrolysis as an approach to treat single-use disposable waste in an environmentally friendly way.

## 2. Materials and Methods

### 2.1. Waste Feedstock and Characterization

Single-use food containers and corrugated fiberboard were collected in a waste recycling store located in Suwon, Gyeonggi, Korea. The two waste types were cleaned by washing or by air blowing to remove impurities and were then cut into slabs (the size of each was 1 cm × 1 cm), as pictured in Figure S1.

Thermogravimetric analysis (TGA) of the two waste feedstocks were conducted by heating the sample from 30 °C and 900 °C with a heating rate of 10 °C min^−1^ under flowing ultra-high-purity (UHP) N_2_ with a flow rate of 60 mL min^−1^ using a Discovery TGA 55 Thermogravimetric Analyzer (TA Instruments, New Castle, DE, USA). The procedures for the proximate and ultimate analyses of the two feedstocks are given in Supporting Information.

### 2.2. Pyrolyzer Setup, Experimental Procedure, and Pyrolytic Product Analysis

A pyrolyzer system was built for co-pyrolysis experiments on single-use food containers and corrugated fiberboard, which is schematically depicted in Figure S2. A quartz tube (length: 0.6 m; outside diameter: 25 mm; inside diameter: 21 mm), a temperature-controllable hinged tube furnace, a mass flow controller (MFC), and a condenser composed of an ice trap maintained at −1 °C and a dry ice/acetone trap maintained at −50 °C compose the reactor setup.

The feedstock (single-use food container slabs or corrugated fiberboard slabs) of 1 g was centered in the quartz tube. For the pyrolysis of a mixture of single-use food containers and corrugated fiberboard, the single-use food container slabs of 0.5 g and the corrugated fiberboard slabs of 0.5 g (total 1 g) were used as the feedstock. The feedstock-loaded quartz tube was heated by the tube furnace. The flow rate of N_2_ (UHP grade) was controlled by the MFC. The sample injector of a Fusion Gas Analyzer micro-gas chromatograph (micro-GC) (INFICON (Bad Ragaz, Switzerland) was directly connected to the outlet of the reactor to ensure that the pyrolytic gas samples were being directly and regularly injected into the micro-GC. Each experiment was performed thrice to check the reproducibility of collected data.

The micro-GC equipped with thermal conductivity detector was used to analyze non-condensable gases. Information about the column used for the micro-GC analysis and the relevant analysis conditions are provide in Table S1. A five-point external standard calibration using standard gas mixtures was applied to the micro-GC analysis.

A GC instrument equipped with a mass spectrometry (GC/MS) (GC model: 8890; MS model: 5977B) manufactured by Agilent Technologies (Santa Clara, CA, USA) was used to identify the condensable species collected in the condenser and to determine their concentrations. Table S2 provides the information regarding the column used for the GC/MS analysis and the relevant analysis conditions. The National Institute of Standards and Technology (NIST) mass spectral library was referred to in order to identify condensable species, and an internal standard method using 5-methylfurfural (10 ng mL^−1^) was applied to determine their concentrations.

## 3. Results and Discussion

### 3.1. Feedstock Characterization

Table 2 summarizes the results of the proximate and ultimate analyses (dry basis) for the single-use food containers and corrugated fiberboard. The single-use food containers were mostly composed of volatile species (98.4 wt.%) with minimal residue formation (i.e., ash). They did not contain oxygen, nitrogen, or sulfur because single-use food containers are made of polypropylene ((C_3_H_6_)*_n_*). The corrugated fiberboard was composed of 72.3 wt.% volatile matter, 14.6 wt.% fixed matter, and 11.6 wt.% ash. It also contained moisture (1.5 wt.%), even at a dry basis because typical corrugated fiberboard contains a certain range of moisture (about 5–10%) to properly maintain its compression strength. It is often composed of 37.5 wt.% carbon, 5.1 wt.% hydrogen, and 33.8 wt.% oxygen. No nitrogen or sulfur was detected in the ultimate analysis of the corrugated fiberboard.

**Figure 1:**
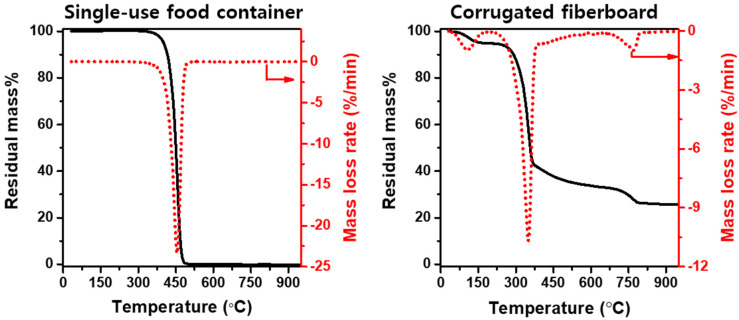
Residual mass% and mass loss rate of the single-use food containers and those of the corrugated fiberboard.

Figure 1 represents thermal mass loss and mass loss rate curves obtained via the TGA of the single-use food containers and the corrugated fiberboard. As presented in Figure 1, a single degradation zone is observed for thermal degradation of the single-use food containers, which indicates that the single-use food containers were completely thermally degraded between 300 °C and 500 °C. Figure 1 shows three distinctive thermal degradation zones for the thermal degradation of the corrugated fiberboard. The first zone, between 30 °C and 140 °C, is associated with the evaporation of residual moisture. The second zone demonstrates a large peak in the mass loss rate curve ranging from 230 °C to 380 °C, corresponding to the volatilization of the majority of the sample mass (~55 wt.%). This is well consistent with the volatile matter content of the corrugated fiberboard shown in Table 1. The final zone started at approximately 380 °C and finished at approximately 650 °C, which generated a tail slowly approaching a mass loss rate of zero as the sample was pyrolyzed to yield a solid residue. The solid residue in the amount of approximately 24 wt.% of the initial sample mass corresponds to the ash content of the corrugated fiberboard (Table 2).

### 3.2. Pyrolysis of Single-Use Food Container, Corrugated Fiberboard, and Their Mixture

**Figure 2:**
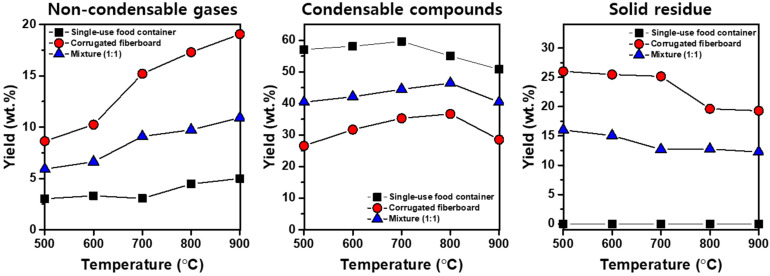
Yields of the pyrolytic products produced via the pyrolysis of single-use food containers, corrugated fiberboard, and a mixture of single-use food containers and corrugated fiberboard (1:1; mass basis) as a function of pyrolysis temperature.

Figure 2 presents the yields of the products produced via the pyrolysis of the single-use food containers, corrugated fiberboard, or a 1:1 mixture of single-use food containers and corrugated fiberboard. For the single-use food container pyrolysis, the yield of condensable compounds was higher than the yield of non-condensable gases. This should be because of the high molecular weight of the polymer of which the single-use food containers were made, given relatively the very low molecular weights of non-condensable gases. The yield of non-condensable gases decreased in an order of corrugated fiberboard > single-use food container/corrugated fiberboard mixture > single-use food containers. This is likely because of the content of cellulose and hemicellulose in the corrugated fiberboard, considering that the formation of non-condensable gases during biomass pyrolysis is susceptible to the content of cellulose and hemicellulose [30]. For the three feedstocks, the yield of non-condensable gases increased by increasing the pyrolysis temperature from 500 to 900 °C, which can be ascribed to thermal cracking of heavy molecules to light molecules (e.g., non-condensable gases), which is enhanced at higher temperatures.

For the single-use food container pyrolysis, the yield of the condensable compounds was highest at 700 °C, which then decreased with an increase in the temperature to 900 °C (Figure 2). Similar to the case of the single-use food container pyrolysis, the yield of the condensable compounds obtained via the pyrolysis of the corrugated fiberboard and the single-use food container/corrugated fiberboard mixture increased to >700 °C and then decreased when the temperature further increased to 900 °C. This observation should be associated with the condensable pyrolysis vapors that are thermally cracked more readily at temperatures higher than >700 °C [31,32].

Figure 2 also shows that no residual solid existed after the single-use food container pyrolysis was done. This is an indication of a complete thermal degradation of the single-use food container at > 500 °C, as found in its TGA results (Figure 1). However, the pyrolysis of the corrugated fiberboard or the single-use food container/corrugated fiberboard mixture led to solid residue, meaning that solid residue originates from the corrugated fiberboard. The increase in the pyrolysis temperature decreased the yield of solid residue for the pyrolysis of both feedstocks. For example, the yield of the solid residue after the corrugated fiberboard pyrolysis decreased from 26.0 wt.% to 19.3 wt.% as the pyrolysis temperature rose from 500 °C to 900 °C. This is because pyrolysis vapors release from the solid phase feedstock more easily [33], which is attributed to the cleavage of the O–H and C–H bonds and is promoted by increasing the pyrolysis temperature [34].

**Figure 3:**
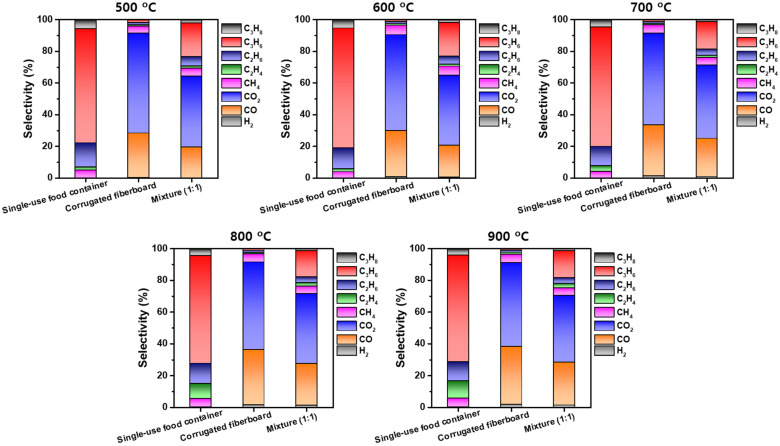
Selectivities toward non-condensable gases contained in the pyrolytic gas produced via the pyrolysis of single-use food containers, corrugated fiberboard, and a mixture of single-use food containers and corrugated fiberboard (1:1; mass basis) at different pyrolysis temperatures.

The pyrolysis of the three feedstocks resulted in the production of non-condensable gases including hydrogen (H_2_), carbon monoxide (CO), carbon dioxide (CO_2_), methane (CH_4_), ethylene (C_2_H_4_), ethane (C_2_H_6_), propylene (C_3_H_6_), and propane (C_3_H_8_). Figure 3 presents the selectivities toward the eight non-condensable gases produced via the pyrolysis of single-use food containers, corrugated fiberboard, and their 1:1 mixture at different temperatures. The C_3_H_6_ accounts for about 67–76% of the single-use food container-derived pyrolytic gas, resulting from the depolymerization of polypropylene from which the disposable food containers were made. For the single-use food container pyrolysis, the C_2_H_4_ could be produced via the thermal disproportionation of C_3_H_6_ [35]. The increase in the pyrolysis temperature decreased the selectivity toward C_3_H_6_ and increased the selectivity toward C_2_H_4_. This would be because the thermal C_3_H_6_ disproportionation is promoted at elevated temperatures. Dehydrogenation reactions that took place during the pyrolysis lead to H_2_ [36]. The H_2_ could also react with C_2_H_4_ and C_3_H_6_, resulting in C_2_H_6_ and C_3_H_8_, respectively. The CH_4_ is generated via thermal cracking of pyrolytic volatiles [37]. No CO and CO_2_ were found in the single-use food container-derived pyrolytic gas because the single-use food containers did not contain any oxygen source (Table 2). The single-use food container-derived pyrolytic gases produced at different temperatures had similar higher heating values (HHVs) of approximately 50 MJ kg^−1^ because their compositions were independent on the pyrolysis temperature. Note that the HHVs were calculated using the heat of combustion and the yields of the non-condensable gases.

Unlike the case of the single-use food container pyrolysis, the pyrolytic gas that evolved from the corrugated fiberboard was mostly composed of CO and CO_2_, attributed to its high oxygen content (33.8 wt.%; Table 1). The selectivity toward CO increased from 28.2% to 36.4% with the increase in the pyrolysis temperature from 500 °C to 900 °C, while the selectivity toward CO_2_ decreased from 63.1% to 52.9%. More H_2_ was released at higher temperatures because dehydrogenation reactions are facilitated at high temperatures for biomass pyrolysis [38]. The CO_2_ evolved while the pyrolysis of the corrugated fiberboard reacted with the H_2_ to form CO [39] via reverse water–gas shift reaction more favored by higher temperatures [40]. The selectivities toward CH_4_, C_2_H_4_, C_2_H_6_, C_3_H_6_, and C_3_H_8_ were not affected by the change in pyrolysis temperature as much as those toward CO and CO_2_ were. The corrugated fiberboard-derived pyrolytic gas had HHVs ranging from 7.6 MJ kg^−1^ to 11.1 MJ kg^−1^. The HHVs of the corrugated fiberboard-derived pyrolytic gases were a lot lower than those of the single-use food container-derived pyrolytic gases, owing to high contents of non-combustible gas (i.e., CO_2_) in the corrugated fiberboard-derived pyrolytic gases (Figure 3).

When co-feeding single-use food containers with corrugated fiberboard, the selectivities toward CO and CO_2_ was greatly enhanced compared with the single-use food container pyrolysis because the corrugated fiberboard pyrolysis resulted in the pyrolytic gas mostly comprising CO and CO_2_ (Figure 3). The selectivities toward CO and CO_2_ were proportional to the corrugated fiberboard loading at all tested temperatures. This was due to both the high oxygen content of the corrugated fiberboard (33.8 wt.%) and the fact that there was no oxygen in the single-use food containers (Table 2). However, the selectivities toward C_1_–C_3_ hydrocarbons are inversely proportional to the corrugated fiberboard loading at all tested temperatures. Considering that C_1_–C_3_ hydrocarbons primarily originate from the polypropylene that constitutes the single-use food containers, co-feeding corrugated fiberboard led to lower selectivities toward C_1_–C_3_ hydrocarbons than the pyrolysis of the single-use food containers. In addition, the pyrolysis of the single-use food container/corrugated fiberboard mixture led to an increase in the selectivity toward H_2_ at all of the temperatures that were tested, compared to the pyrolysis of the single feedstock. For instance, at 800 °C, the pyrolysis of the single-use food container/corrugated fiberboard mixture gave a 1.4% selectivity toward H_2_ (mass basis), while the pyrolysis of the single-use food containers gave a 0.2% selectivity toward H_2_. This can be explained by two mechanisms. First, the single-use food containers act as a hydrogen donor [41], which can be attributed to its higher H content than that of the corrugated fiberboard (Table 2) [42]. Second, water evaporated during the corrugated fiberboard pyrolysis serves as a reactive species that enhances thermal decomposition and the dehydrogenation of pyrolysis volatiles that are evolved from the single-use food containers [43]. The H_2_ selectivity achieved by the co-pyrolysis of single-use food containers and corrugated fiberboard was comparable with those achieved by co-pyrolysis of other feedstocks. For example, at 700 °C, the co-pyrolysis of wood bark and food waste (1:1 mixture) resulted in 2.2% H_2_ selectivity [25]. When pyrolyzing herbal medicine waste with food waste with a ratio of 50:50 at 700 °C, it led to 1.9% H_2_ selectivity [24]. The co-pyrolysis of a 1:1 mixture of face mask and food waste gave 1.6% H_2_ selectivity at 700 °C [23]. At a comparable temperature and feedstock composition, the co-pyrolysis of single-use food containers and corrugated fiberboard exhibited 1.2% H_2_ selectivity (this study).

The condensable compounds listed in Table S3 were observed in the pyrolytic liquid (i.e., the mixture of condensable compounds) created by the single-use food containers, corrugated fiberboard, or the single-use food container/corrugated fiberboard mixture. The selectivities toward each chemical group are shown in Table 3. The pyrolysis of the single-use food containers led to the pyrolytic liquid composed of C_7_–C_32_ hydrocarbons without phenolic compounds, polycyclic aromatic compounds, and oxygenates. No oxygenates were formed because the single-use food containers did not contain oxygen (Table 2). Considering that the C_7_–C_32_ hydrocarbons are in a range products including gasoline, jet fuel, diesel, and motor oil [44], pyrolysis has the potential to upcycle single-use food containers to make fuel-range chemicals. The difference between the selectivities toward the hydrocarbons obtained at different pyrolysis temperatures was not significant.

Unlike the case of the single-use food container pyrolysis, the condensable compounds found in the pyrolytic liquid made from the corrugated fiberboard was moslty composed of phenolic compounds and oxygenates. Given that corrugated fiberboard typically contains cellulose and hemicellulose [45], oxygenates including phenolic compounds, alcohols, acids, aldehydes, ketones, esters, acetates, and polycyclic aromatic compounds should be formed by the pyrolysis of the cellulosic and hemicellulosic portions [46] of the corrugated fiberboard. Table 3 shows the selectivities toward condensable compounds according to classification. The difference between the selectivities achieved between 500 °C and 900 °C was not significant.

Table 3 also summarizes the selectivities toward the groups of condensable compounds observed in the pyrolytic liquid derived from the mixture of the single-use food containers and corrugated fiberboard. The C_7_–C_32_ hydrocarbon distribution created by the mixture was similar to that made from the single-use food containers. When mixing the single-use food containers with the corrugated fiberboard, the selectivity toward oxygenates decreased compared to the pyrolysis of the corrugated fiberboard only. In addition, the co-pyrolysis of the single-use food containers and corrugated fiberboard resulted in no phenolic compounds or polycyclic aromatic compounds (0% selectivity), while significant amounts of phenolic compounds and polycyclic aromatic compounds were found (>20% selectivity) in the corrugated fiberboard-derived pyrolytic liquid. Although other co-pyrolysis processes (e.g., wood bark/food waste [25], herbal medicine waste/food waste [24], and face mask/food waste [23]) have been reported to suppress the formation of such compounds, those processes resulted in a pyrolytic product containing certain amounts of phenolic compounds and polycyclic aromatic compounds. This should indicate that the addition of the single-use food containers suppresses the formation of phenolic compounds and polycyclic aromatic compounds during the pyrolysis of the corrugated fiberboard. It would be applicable to use pyrolysis as an environmentally friendly approach to cleanly dispose of single-use disposable wastes via pyrolysis because the phenolic compounds [47] and the polycyclic aromatic compounds [48] are hazardous environmental pollutants with carcinogenicity and cause the formation of particulate matter in the air [49].

### 3.3. Practical Implications of This Study

Even though this study showed that the co-pyrolysis of single-use food containers and corrugated fiberboard is effective at valorizing these types of single-use disposable waste without the formation of hazardous species, the effects of the co-pyrolysis parameters other than temperature and feedstock composition, such as feedstock loading and flow rate of pyrolysis agent, were not explored. Therefore, such parameters need to be fully investigated in future studies. In addition, the co-pyrolysis of the single-use disposable waste with other types of waste such as organic waste would be required to maximize the effectiveness of the upgrading of single-use waste. From a process point of view, the co-pyrolysis of single-use food containers and corrugated fiberboard could be conducted under microwave heating to maximize the heating efficiency of the pyrolysis process. Investigation of the sustainability features of the lab-scale experimental results via life cycle assessment, exergy, and their combinations should also be considered as future research.

The waste collection is a major part of municipal solid waste management, the costs of which are highly associated with legislation [50]. Thus, in addition to the technological development of the co-pyrolysis of single-use disposable waste, government legislation and society need induce the employment of the co-pyrolysis process in the waste management sector. To make the co-pyrolysis process a more sustainable approach to treat single-use disposable waste, the sustainable roadmaps for the collection, separation, and transportation of single-use disposable waste to co-pyrolysis facilities and relevant rules and legislation need to be established.

## 4. Conclusions

In this study, the pyrolysis of single-use food containers and corrugated fiberboard was studied to make an effort to devise a strategy for the clean waste disposal of municipal plastic waste. The following conclusions can be reached:

- Pyrolysis of single-use food containers:

The pyrolysis of the single-use food containers mostly produced pyrolytic liquid (~60 wt.%), and no solid residue was found between 500 °C and 900 °C. The pyrolytic product derived from the single-use food containers consisted of C_1_–C_32_ hydrocarbons with no oxygenated compounds or polycyclic aromatic compounds, mostly because the single-use food containers contained no oxygen. The selectivities toward the chemical compounds contained in the single-use food container-derived pyrolytic product obtained at different pyrolysis temperatures were not significantly different. About 20–26 wt.% of the corrugated fiberboard remained as solid after its pyrolysis.

- Pyrolysis of corrugated fiberboard:

The pyrolytic gas derived from the corrugated fiberboard was composed mostly of CO and CO_2_, while that derived from the single-use food containers was mostly composed of C_1_–C_3_ hydrocarbons. The corrugated fiberboard-derived pyrolytic liquid contained chemical compounds that can be grouped as phenolic compounds, polycyclic aromatic compounds, and a range of oxygenates including acids, alcohols, aldehydes, ketones, acetates, esters. The selectivity toward each group was not sensitive to pyrolysis temperature.

- Co-pyrolysis of single-use food container and corrugated fiberboard:

The pyrolysis of the single-use food containers by co-feeding with the corrugated fiberboard not only resulted in an enhanced H_2_ production but also in no formation of hazardous environmental pollutants such as phenolic compounds and polycyclic aromatic compounds. To better understand the co-feeding effects on the pyrolysis of single-use food containers and corrugated fiberboard, mechanistic modeling of the pyrolysis system should be performed.

## Figures and Tables

**Table 1:** Effect of co-pyrolysis for the treatment of various wastes.

Feedstock	Pyrolysis Temperature	Co-Pyrolysis Effect	Ref.
Face mask/food waste (25/75)	700 °C	Higher H_2_ selectivity and lower polycyclic aromatic compound (PAC) selectivity than pyrolysis of face mask only	[23]
Food waste/herbal medicine byproduct (25/75)	700 °C	Higher H_2_ selectivity and lower PAC selectivity than pyrolysis of single feedstock	[24]
Wood bark/food waste (50/50)	700 °C	Higher H_2_ selectivity and lower PAC selectivity than pyrolysis of single feedstock	[25]
High-density polyethylene (HDPE)/waste newspaper	400–500 °C	Significant increase in oil phase compared to theoretical yield	[26]
Spent plastic mulch film/swine manure	500 °C	Higher heating value of pyrolytic gas than that of natural gas	[27]
Polystyrene/palm shell (60/40)	600 °C	Maximum liquid yield of 68 wt.% with a high heating value of 40.3 MJ kg^−1^	[28]
HDPE/potato waste	400–500 °C	Enhancement of quantity and quality of pyrolytic liquid with reduced oxygen content	[29]

**Table 2:** Results of proximate and ultimate analysis of the single-use food containers and corrugated fiberboard (unit: wt.%).

**Element**	**Single-Use Food Container**	**Corrugated Fiberboard**
C	84.8	37.5
H	14.2	5.1
O	N.D.	33.8
N	N.D.	N.D.
S	N.D.	N.D.
Ash	1.0	23.6
Total	100	100
**Component**	**Single-Use Food Container**	**Corrugated Fiberboard**
Moisture	0.1	5.5
Volatile matter	98.4	56.3
Fixed matter	0.5	14.6
Ash	1.0	23.6
Total	100	100

**Table 3:** Selectivity (%) toward each group of condensable compounds contained in the pyrolytic liquid (i.e., the mixture of condensable compounds) produced via the pyrolysis of single-use food containers, corrugated fiberboard, and a mixture of single-use food containers and corrugated fiberboard (1:1; mass basis) at different pyrolysis temperatures.

Temperature (°C)	500			600			700			800			900		
Feedstock	Single-Use Food Container	Corrugated Fiberboard	Mixture (1:1)	Single-Use Food Container	Corrugated Fiberboard	Mixture (1:1)	Single-Use Food Container	Corrugated Fiberboard	Mixture (1:1)	Single-Use Food Container	Corrugated Fiberboard	Mixture (1:1)	Single-Use Food Container	Corrugated Fiberboard	Mixture (1:1)
C_7_	0	0.1	0	0	0.2	0	0	0.2	0	0	0.2	0	0	0.1	0
C_8_	0	1.6	0	0	1.6	0	0	1.6	0	0	1.7	0	0	1.1	0
C_9_	0.5	0	0.1	0.4	0	0.1	0.6	0	0.2	0.6	0	0.1	0.4	0	0.2
C_10_	4.3	0.8	5.0	4.4	0.8	5.1	4.7	0.7	5.2	4.3	0.7	4.9	4.5	0.8	5.2
C_11_	1.1	0.1	0	1.0	0.3	0	1.1	0.2	0	1.1	0.3	0	1	1.1	0
C_12_	17.6	0.2	12.3	17.5	0.2	12.6	17.5	0.2	12.9	17.6	0.4	12.4	18	0.4	12.4
C_13_	1.4	0	0	1.4	0	0	1.6	0	0	1.4	0	0	1.6	0	0
C_14_	3.0	0	1.3	2.7	0	1.2	3.0	0	1.4	2.7	0	1.4	2.6	0	1.5
C_15_	4.7	1.6	0	5.0	1.6	0	5.0	1.6	0	5	1.7	0	4.7	1.7	0
C_17_	0.1	0	0.6	0.2	0	0.6	0.2	0	0.6	0.2	0	0.7	0.1	0	0.7
C_18_	0.5	0.5	0	0.4	0.4	0	0.5	0.2	0	0.4	0.5	0	0.4	0.4	0
C_19_	4.1	0	1.3	4.2	0	1.3	4.2	0	1.5	4	0	1.3	4.3	0	1.2
C_20_	16.8	0	7.1	16.8	0	7.1	16.8	0	7.2	16.9	0	7.1	17.1	0	7.3
C_22_	3.6	0	0	3.6	0	0	3.3	0	0	3.7	0	0	3.4	0	0
C_23_	3.2	0	0	3.3	0	0	3.5	0	0	3.6	0	0	3.3	0	0
C_24_	7.9	0.6	0	7.8	0.5	0	7.7	0.5	0	7.6	0.4	0	7.9	0.4	0
C_25_	7.6	0	9.4	7.5	0	9.3	7.6	0	9.6	7.7	0	9.7	7.8	0	9.8
C_26_	13.9	0	1.8	13.9	0	1.7	13.3	0	1.7	13.5	0	1.5	13.2	0	1.5
C_27_	0.5	0	0	0.5	0	0	0.4	0	0	0.6	0	0	0.3	0	0
C_29_	0.7	0	0	0.6	0	0	0.7	0	0	0.7	0	0	0.7	0	0
C_32_	8.5	0	2.1	8.8	0	2.4	8.3	0	2.3	8.4	0	2.3	8.7	0	2.4
Phenolic compounds	0	19.7	0	0	21.0	0	0	21.2	0	0	20.4	0	0	20.8	0
Polycyclic aromatic compounds	0	1.0	0	0	0.7	0	0	1.1	0	0	1.0	0	0	1.0	0
Oxygenates ^1^	0	73.8	59.0	0	72.7	58.6	0	72.5	57.4	0	72.7	58.6	0	72.2	57.8

^1^ Including alcohols, aldehydes, ketones, acids, acetates, and esters.

## Supplementary Materials

The following are available online at https://www.mdpi.com/article/10.3390/polym13162617/s1, Figure S1: Single-use food container slabs and corrugated fiberboard slabs used as the feedstock in this study, Figure S2: Schematic of the reactor setup used in this study, Table S1: Micro-GC setting for the analysis of non-condensable gases, Table S2: GC/MS setting for the analysis of condensable compounds, Table S3: Condensable compounds in the pyrolytic liquid derived from the single-use food containers and corrugated fiberboard identified by the GC–MS analysis.
