# Supplementary material for: Single-Use Disposable Waste Upcycling via Thermochemical Conversion Pathway
Source: Polymers (Basel). 2021 Aug 6;13(16):2617. doi: 10.3390/polym13162617 (PMC8400630; doi:10.3390/polym13162617)
Supplement: Supplementary file 1 [file polymers-13-02617-s001.zip › polymers-1333251-supplementary.pdf]

Supplementary Materials

Single-Use Disposable Waste Upcycling via Thermochemical Conversion Pathway

Junghee Joo [1,†], Seonho Lee [2,†], Heeyoung Choi [2,†], Kun-Yi Andrew Lin [3] and Jechan Lee [1,2,*]

[1] Department of Energy Systems Research, Ajou University, 206 World cup-ro, Suwon 16499, Korea; jurno@ajou.ac.kr
[2] Department of Environmental and Safety Engineering, Ajou University, 206 World cup-ro, Suwon 16499, Korea; idosunho99@ajou.ac.kr (S.L.); chk6788@ajou.ac.kr (H.C.)
[3] Innovation and Development Center of Sustainable Agriculture, Department of Environmental Engineering, National Chung Hsing University, 250 Kuo-Kuang Road, Taichung 402, Taiwan; linky@nchu.edu.tw
* Correspondence: jlee83@ajou.ac.kr
† Designates the co-first authors who contributed equally to this work.

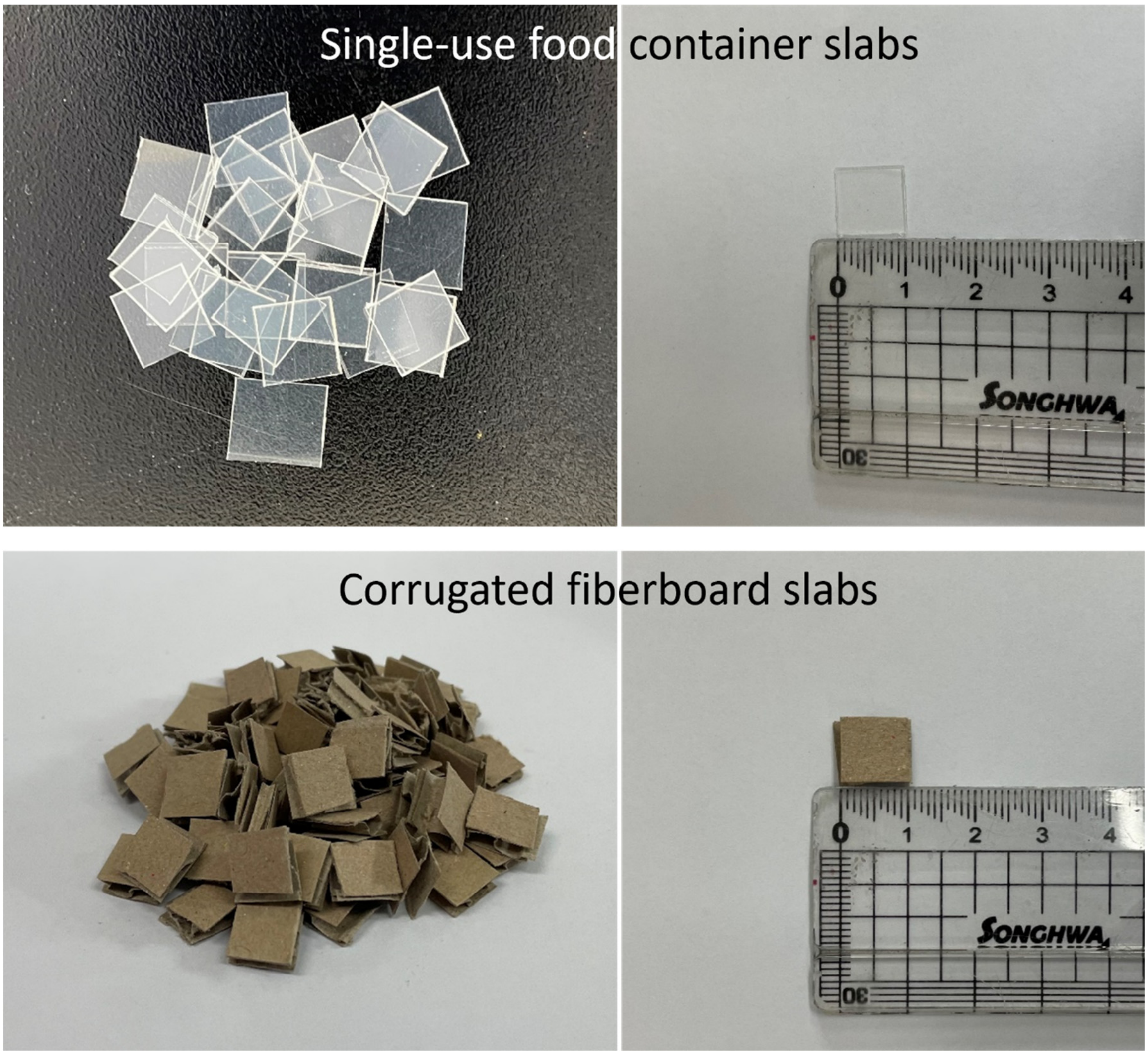

Figure S1. Single-use food container slabs and corrugated fiberboard slabs used as the feedstock in this study

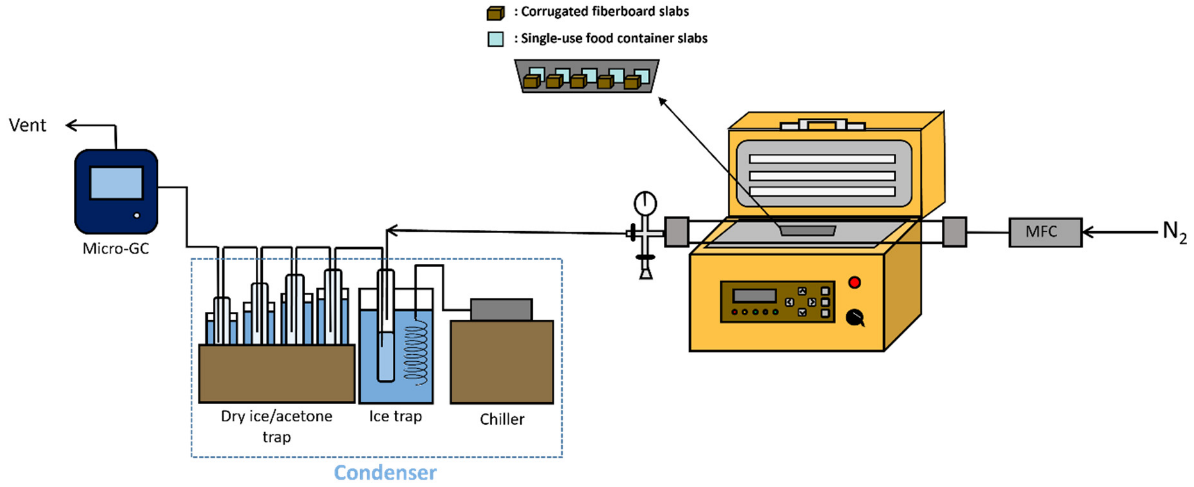

Figure S2. Schematic of the reactor setup used in this study

Table S1. Micro-GC setting for the analysis of non-condensable gases

Module		Module A	Module B
Column		Rt-Molsieve 5A	Rt-Q-Bond
Sample pump setting	Mode	Continuous	Continuous
	Pumping time	15 s	15 s
Column setting	Carrier gas	Argon (≥99.999%)	Helium (≥99.999%)
	Column pressure	20 psi	17 psi
	Initial temperature	50 °C (40 s)	50 °C (30 s)
	Ramping time	50 s	60 s
	Final temperature	100 °C (40 s)	110 °C (40 s)
	Total analysis time	130 s	130 s
Injector setting	Temperature	90 °C	90 °C
	Injection time	30 ms	30 ms
TCD setting	Temperature	70 °C	70 °C
	Data rate	50 Hz	50 Hz

Table S2. GC/MS setting for the analysis of condensable compounds

Column	HP-5MS Ultra Inlet column (0.25 mm × 0.25 um × 30 m)	
Oven setting	Initial temperature	35 °C (1 min)
	Ramping	3 °C min^{-1}
	Final temperature	290 °C (4 min)
	Total analysis time	90 min
Column setting	Column flow	1 mL min^{-1}
	Carrier gas	Helium (≥99.999%)
	Carrier gas flow rate	3 mL min^{-1}
Injector setting	Injection mode	Splitless
	Injection volume	1 μL
	Temperature	280 °C
MS setting	m/z range	35~550 amu
	Aux temperature	300 °C

Table S3. Condensable compounds in the pyrolytic liquid derived from the single-use food container and corrugated fiberboard, identified by the GC–MS analysis

Chemical compound	Chemical formula
Phenolic compounds	
(E)-3-(4-hydroxy-2-methoxyphenyl)acrylaldehyde	C10H10O3
4-hydroxy-3,5-dimethoxybenzaldehyde	C9H10O4
(E)-3-(4-hydroxy-3,5-dimethoxyphenyl)acrylaldehyde	C11H12O4
(E)-2,6-dimethoxy-4-(prop-1-enyl)phenol	C11H14O3
(E)-2-methoxy-4-(prop-1-enyl)phenol	C10H12O2
(E)-2-methoxy-5-(prop-1-enyl)phenol	C10H12O2
(E)-4-(3-hydroxyprop-1-enyl)-2,6-dimethoxyphenol	C11H14O4
1-(3-hydroxy-4-(3,4,5-trihydroxy-6-(hydroxymethyl)tetrahydro-2H-pyran-2-yloxy)phenyl)ethanone	C14H18O8
1-(4-hydroxy-3-methoxyphenyl)propan-2-one	C10H12O3
2,3-dimethylphenol	C8H10O
2,5-dimethylphenol	C8H10O
2,6-dimethoxy-4-vinylphenol	C10H12O3
2,6-dimethoxyphenol	C8H10O3
2,6-dimethylphenol	C8H10O
2-methoxy-4-methylphenol	C8H10O2
2-Methoxy-4-vinylphenol	C9H10O2
2-methoxyphenol	C7H8O2
2-tert-butyl-4-methoxyphenol	C11H16O2
3-(hydroxymethyl)-5-methoxyphenol	C8H10O3
3-hydroxy-4-methoxybenzaldehyde	C8H8O3
3-methylbenzene-1,2-diol	C7H8O2
4-(hydroxymethyl)phenol	C7H8O2
4-allyl-2,6-dimethoxyphenol	C11H14O3
4-ethyl-2-methoxyphenol	C9H12O2
4-methylbenzene-1,2-diol	C7H8O2
5-allyl-2-methoxyphenol	C10H12O2
5-tert-butylbenzene-1,2,3-triol	C10H14O3
6-hydroxychroman-2-one	C9H8O3
m-cresol	C7H8O
p-Cresol	C7H8O
Phenol	C6H6O
pyrocatechol	C6H6O2
Resorcinol	C6H6O2
Oxygenates	

(1R,4R)-3,3,4-trimethyl-4-p-tolylcyclopentanol	$C_{15}H_{22}O$
(2-(5,5-dimethoxypent-1-en-2-yl)-5-methylcyclopentyl)methanol	$C_{14}H_{26}O_3$
(2,4,6-trimethylcyclohexyl)methanol	$C_{10}H_{20}O$
(2-allyl-3,6-dimethoxyphenyl)methanol	$C_{12}H_{16}O_3$
(2R,4R,6R)-2,4,6-trimethyldodec-11-en-1-ol	$C_{15}H_{30}O$
(2Z,4E,6Z,8E)-3,7-dimethyl-9-(2,6,6-trimethylcyclohex-1-enyl)nona-2,4,6,8-tetraen-1-ol	$C_{20}H_{30}O$
(3-Methyl-1,4-diphenylbicyclo[2.2.0]hex-2-yl)methanol	$C_{20}H_{22}O$
(E)-2,2,6-trimethyl-1-(3-methylbuta-1,3-dienyl)-7-oxabicyclo[4.1.0]heptan-3-ol	$C_{14}H_{22}O_2$
(E)-2,4,4,7-tetramethylocta-5,7-dien-3-ol	$C_{12}H_{22}O$
(E)-heptadeca-1,9-dien-4,6-diyn-3-ol	$C_{17}H_{24}O$
(E)-hexadec-2-en-1-ol	$C_{16}H_{32}O$
(tetrahydro-[1,3]dioxino[5,4-d][1,3]dioxine-4,8-diyl)dimethanol	$C_8H_{14}O_6$
(Z)-2-(octadec-9-enyloxy)ethanol	$C_{20}H_{40}O_2$
1-(4-methoxyphenyl)propan-1-ol	$C_{10}H_{14}O_2$
12-Oxa[tetracyclo[5.2.1.1(2,6).1(8,11)]dodecan-10-ol, 3-acetoxy-	$C_{13}H_{18}O_4$
2,10-dimethylundec-9-en-1-ol	$C_{13}H_{26}O$
2-hexyldecan-1-ol	$C_{16}H_{34}O$
2-isopropyl-5-methylheptan-1-ol	$C_{11}H_{24}O$
2-methylhexadecan-1-ol	$C_{17}H_{36}O$
2-octyldecan-1-ol	$C_{18}H_{38}O$
2-octyldodecan-1-ol	$C_{20}H_{42}O$
3,7,11,15-tetramethylhexadecan-1-ol	$C_{20}H_{42}O$
3,7,11-trimethyldodecan-1-ol	$C_{15}H_{32}O$
5-(prop-2-ynyloxy)pentan-2-ol	$C_8H_{14}O_2$
5-isopropyl-6-methylhept-5-en-3-yn-2-ol	$C_{11}H_{18}O$
but-3-en-2-ol	C_4H_8O
cyclododecylmethanol	$C_{13}H_{26}O$
docosan-1-ol	$C_{22}H_{46}O$
heptacosan-1-ol	$C_{27}H_{56}O$
nonadecan-1-ol	$C_{19}H_{40}O$
octacosan-1-ol	$C_{28}H_{58}O$
prop-2-en-1-ol	C_3H_6O
tetracosan-1-ol	$C_{24}H_{50}O$
Tricosan-2-ol	$C_{23}H_{48}O$
β-D-Glucopyranose, 1,6-anhydro-	$C_6H_{10}O_5$
(2R,2'R,3S,3'S,4S,4'S,5R,5'R,6R,6'R)-6,6'-((2S,3S,4R,5R)-4-hydroxy-2,5-bis(hydroxymethyl)tetrahydrofuran-2,3-diyl)bis(oxy)bis(2-(hydroxymethyl)tetrahydro-2H-pyran-3,4,5-triol)	$C_{18}H_{32}O_{16}$
(S)-2-hydroxy-2-((R)-3-hydroxy-5-oxo-4-(palmitoyloxy)-2,5-dihydrofuran-2-yl)ethyl palmitate	$C_{38}H_{68}O_8$

1-(furan-2-yl)-2-hydroxyethanone	$C_6H_6O_3$
2-Hydroxy-2-(5-methylfuran-2-yl)1-phenylethanone	$C_{13}H_{12}O_3$
3,5-dimethylfuran-2(5H)-one	$C_6H_8O_2$
3-methylfuran	C_5H_6O
4-hydroxy-2,5-dimethylfuran-3(2H)-one	$C_6H_8O_3$
4-Hydroxy-4a,8-dimethyl-3-methylene-3,3a,4,4a,7a,8,9,9a-octahydroazuleno[6,5-b]furan-2,5-dione	$C_{15}H_{18}O_4$
4-hydroxy-5-methylfuran-3(2H)-one	$C_5H_6O_3$
5-(hydroxymethyl)furan-2-carbaldehyde	$C_6H_6O_3$
5-heptyldihydrofuran-2(3H)-one	$C_{11}H_{20}O_2$
5-methylfuran-2(5H)-one	$C_5H_6O_2$
9-methyl-3-methyleneoctahydro-2H-spiro[azuleno[4,5-b]furan-6,2'-oxirane]-2,8(9bH)-dione	$C_{15}H_{18}O_4$
furan-2(5H)-one	$C_4H_4O_2$
furan-2-carbaldehyde	$C_5H_4O_2$
furan-2-ylmethanol	$C_5H_6O_2$
furan-3-carbaldehyde	$C_5H_4O_2$
α,β-Gluco-octonic acid lactone	$C_8H_{14}O_8$
(E)-2-butylidenesuccinic acid	$C_8H_{12}O_4$
(E)-but-2-enoic acid	$C_4H_6O_2$
(E)-pent-2-enoic acid	$C_5H_8O_2$
(E)-undec-2-enoic acid	$C_{11}H_{20}O_2$
(Z)-docos-13-enoic acid	$C_{22}H_{42}O_2$
2,3-Dimethylfumaric acid	$C_6H_8O_4$
3-Methyloxirane-2-carboxylic acid	$C_4H_6O_3$
Gibbane-1,10-dicarboxylic acid, 4a-(hydroxymethyl)-1-methyl-8-methylene-, 1,4a-lactone, 10-methyl ester, (1α,4aα,4bβ,10β)-	$C_{21}H_{28}O_4$
icosa-5,8,11,14-tetraynoic acid	$C_{20}H_{24}O_2$
palmitic acid	$C_{16}H_{32}O_2$
Propanoic acid	$C_3H_6O_2$
(2E,4E,6E)-ethyl 9-formyl-10-oxodeca-2,4,6,8-tetraenoate	$C_{13}H_{14}O_4$
(E)-(3a,10a-dihydroxy-2,10-dimethyl-3,8-dioxo-3,3a,4,6a,7,8,9,10,10a,10b-decahydrobenzo[e]azulen-5-yl)methyl acetate	$C_{19}H_{24}O_6$
(Z)-13,13-dimethyltetradec-11-enyl acetate	$C_{18}H_{34}O_2$
11,13-dimethyltetradec-12-enyl acetate	$C_{18}H_{34}O_2$
3,7,11,15-tetramethylhexadecyl acetate	$C_{22}H_{44}O_2$
2-oxobutyl acetate	$C_6H_{10}O_3$
2-oxopropyl acetate	$C_5H_8O_3$
(2E,6E)-3,7-dimethylnona-2,6-dienal	$C_{11}H_{18}O$
2-((1E,3E,5E)-4-methyl-6-(2,6,6-trimethylcyclohex-1-enyl)hexa-1,3,5-trienyl)cyclohex-1-enecarbaldehyde	$C_{23}H_{32}O$
2,4-dimethylbenzaldehyde	$C_9H_{10}O$

2,5-dimethylbenzaldehyde	C9H10O
4-(3,4-dihydroxy-6-(hydroxymethyl)-5-(3,4,5-trihydroxy-6-(hydroxymethyl)tetrahydro-2H-pyran-2-yloxy)tetrahydro-2H-pyran-2-yloxy)-3-methoxybenzaldehyde	C20H28O13
Benzaldehyde	C7H6O
cyclopentanecarbaldehyde	C6H10O
Pentanal	C5H10O
Succindialdehyde	C4H6O2
2,2'-(methoxymethylene)bis(vinylbenzene)	C18H18O
phenylmethylene dibutyrate	C15H20O4
(S,E)-4-hydroxy-3,5,5-trimethyl-4-(3-oxobut-1-enyl)cyclohex-2-enone	C13H18O3
1,2,4-Metheno-1H-cyclobuta[cd]pentalene-3,5-diol, octahydro-	C10H12O2
2,3-Anhydro-d-mannosan	C6H8O4
2-hydroxycyclopent-2-enone	C5H6O2
2-methylcyclopent-2-enone	C6H8O
3,4-Anhydro-d-galactosan	C6H8O4
3-hydroxycyclohexanone	C6H10O2
5H-Cyclopropa[3,4]benz[1,2-e]azulen-5-one, 9,9a-bis(acetyloxy)-1,1a,1b,4,4a,7a,7b,8,9,9a-decahydro-4a,7b-dihydroxy-1,1,6,8-tetramethyl-3-[(triphenylmethoxy)methyl]-, [1aR-(1aα,1bβ,4aβ,7aα,7bα,8α,9β,9aα)]-	C43H46O8
5-hydroxy-9-oxabicyclo[3.3.1]nonan-2-one	C8H12O3
7,7-Dimethoxy-2,3,4,5,6,7-hexahydro-1H-cyclopenta[a]pentalene	C13H18O2
cyclopentane-1,2-dione	C5H6O2
Bicyclo[2.2.2]octane-1,4-diol	C8H14O2
Tetracyclo[5.3.1.0(2,6).0(8,11)]undecan-4-one, 6-methoxy-2-methyl-	C13H18O2
(E)-(2-phenyl-1,3-dioxolan-4-yl)methyl octadec-9-enoate	C28H44O4
(E)-methyl heptadec-10-en-8-ynoate	C18H30O2
2-ethylhexyl 6-ethyloctan-3-yl phthalate	C26H42O4
ethyl 2,2-dimethyl-5-oxoheptanoate	C11H20O3
ethyl 2-hydroxycyclohexanecarboxylate	C9H16O3
ethyl nona-2,3-dienoate	C11H18O2
methyl 8-(2'-hexylbi(cyclopropan)-2-yl)octanoate	C21H38O2
1-hydroxybutan-2-one	C4H8O2
2,3-dimethylcyclopent-2-enone	C7H10O
3-ethyl-2-hydroxycyclopent-2-enone	C7H10O2
3-methylcyclopentane-1,2-dione	C6H8O2
4-(3,3-dimethyloxiran-2-yl)butan-2-one	C8H14O2
6-methyldihydro-2H-pyran-3(4H)-one	C6H10O2
bi(cyclopentan)-2-one	C10H16O
(2R,3R,4R,5S,6R)-6-(hydroxymethyl)-5-((2R,3R,4S,5S,6R)-3,4,5-trihydroxy-6-(hydroxymethyl)tetrahydro-2H-pyran-2-yloxy)tetrahydro-2H-pyran-2,3,4-triol	C12H22O11

1,4:3,6-Dianhydro-α-d-glucopyranose	C6H8O4
3-hydroxy-2-methyl-4H-pyran-4-one	C6H6O3
5-hydroxy-2-(hydroxymethyl)-4H-pyran-4-one	C6H6O4
6-methyl-3,4-dihydro-2H-pyran-2-one	C6H8O2
α-D-Glucopyranose, 4-O-β-D-galactopyranosyl-	C12H22O11
(2S,3S)-2-hexyl-3-methyloxirane	C9H18O
(E)-4-hydroxy-10-methyl-3,4,7,8,9,10-hexahydro-2H-oxecin-2-one	C10H16O3
1-methoxytetratriacontane	C35H72O
2-(4-methylcyclohex-3-enyl)propan-2-yl formate	C11H18O2
2-(allyloxymethyl)oxirane	C6H10O2
2,3-dihydro-1H-inden-1-one	C9H8O
2-hexyloxirane	C8H16O
Spiro[2.4]heptan-4-one	C7H10O
Polycyclic aromatic compounds	
13-methyl-7,8,9,11,12,13,14,15,16,17-decahydro-6H-cyclopenta[a]phenanthren-17-ol	C18H24O
1-methoxy-4,4a,5,6,7,8-hexahydronaphthalen-2(3H)-one	C11H16O2
(9S,10R,13S,14S)-3-hydroxy-10,13-dimethyl-3,4,9,10,11,12,13,14,15,16-decahydro-1H-cyclopenta[a]phenanthren-17(2H)-one	C19H26O2
1-methyl-9,10-dihydrophenanthrene	C15H14
2-vinylnaphthalene	C12H10
naphthalene	C10H8
C_7	
Toluene	C7H8
(E)-3-allylidenecyclobut-1-ene	C7H8
C_8	
Styrene	C8H8
C_9	
1,3,5-trimethylcyclohexane	C9H18
prop-1-en-2-ylbenzene	C9H10
2,4-dimethylhept-1-ene	C9H18
C_{10}	
2,6,6-trimethylbicyclo[3.1.1]heptane	C10H18
1,5,5-trimethyl-6-methylenecyclohex-1-ene	C10H16
3-methyl-1H-indene	C10H10
(E)-2,2-dimethyloct-3-ene	C10H20
3,3-dimethyloctane	C10H22
(Z)-2,6-dimethylocta-1,6-diene	C10H18
(1R,4R)-1,4-dimethylcyclooctane	C10H20
(1R,4S)-1,4-dimethylcyclooctane	C10H20
C_{11}	

(E)-undec-2-ene	C11H22
bicyclo[4.4.1]undeca-1,3,5,7,9-pentaene	C11H10
4-methyldecane	C11H24
2,6-dimethylnonane	C11H24
2-methyl-3-methylenenonane	C11H22
1,1-dimethyl-2-propylcyclohexane	C11H22
C_{12}	
Biphenyl	C12H10
1-isopropyl-1,3,4-trimethylcyclohexane	C12H24
1-Methyl-2-(4-methylpentyl)cyclopentane	C12H24
4,6,8-trimethylnon-1-ene	C12H24
(E)-2,2-dimethyldec-3-ene	C12H24
(Z)-8-methylundec-2-ene	C12H24
C_{13}	
(E)-tridec-3-ene	C13H26
1,1,6,6-Tetramethylspiro[4.4]nonane	C13H24
C_{14}	
(E)-tetradec-3-ene	C14H28
(E)-tetradec-5-ene	C14H28
5-methylenetridecane	C14H28
2,3,5,8-tetramethyldecane	C14H30
tetradec-1-ene	C14H28
tetradeca-1,13-diene	C14H26
(Z)-tetradec-5-ene	C14H28
C_{15}	
7-isopropyl-1,4a-dimethyldecahydronaphthalene	C15H28
1,3-diphenylpropane	C15H16
1,1,3-trimethyl-2-(3-methylpentyl)cyclohexane	C15H30
(1R,2R)-2-propylbi(cyclohexane)	C15H28
2,6,10-trimethyldodecane	C15H32
C_{17}	
heptadec-1-ene	C17H34
2,6,10-trimethyltetradecane	C17H36
C_{18}	
(E)-octadec-5-ene	C18H36
cyclooctadeca-1,3,5,7,9,11,13,15,17-nonaene	C18H18
p-Terphenyl	C18H14
C_{19}	
nonadec-1-ene	C19H38
2-methyloctadec-7-yne	C19H36

C_{20}	
(E)-icos-3-ene	C20H40
(E)-icos-5-ene	C20H40
(E)-icos-9-ene	C20H40
4-isopropyl-1,7,11-trimethylcyclotetradecane	C20H40
7,11,15-trimethyl-3-methylenehexadec-1-ene	C20H38
(E)-10-methylnonadec-9-ene	C20H40
icos-9-yne	C20H38
icosa-1,19-diene	C20H38
C_{22}	
(3-(2-cyclopentylethyl)pentane-1,5-diyl)dicyclopentane	C22H40
C_{23}	
tricos-1-ene	C23H46
(Z)-tricos-9-ene	C23H46
C_{24}	
tetracos-1-ene	C24H48
1,3,5-triphenylcyclohexane	C24H24
C_{25}	
(Z)-2,6,10,14-tetramethyl-7-(3-methylpent-4-enylidene)pentadecane	C25H48
(4-octyldodecyl)cyclopentane	C25H50
(4-octylheptane-1,7-diyl)dicyclopentane	C25H48
(6-cyclopentyl-3-(3-cyclopentylpropyl)hexyl)cyclohexane	C25H46
hept-1-ene-2,4,6-triyltricyclohexane	C25H44
C_{26}	
hexacos-1-ene	C26H52
C_{27}	
Heptacos-1-ene	C27H54
C_{29}	
Nonacos-1-ene	C29H58
C_{32}	
4-pentyl-4'-(4-(4-propylcyclohexyl)phenyl)bi(cyclohexane)	C32H52
2-((3R)-3-ethyl-3,6,10,13,14-pentamethylpentadecyl)-1,1,3,6-tetramethylcyclohexane	C32H64
